# Vacancy and Doping States in Monolayer and bulk Black Phosphorus

**DOI:** 10.1038/srep14165

**Published:** 2015-09-18

**Authors:** Yuzheng Guo, John Robertson

**Affiliations:** 1 Engineering Dept, Cambridge University, Cambridge, CB2 1PZ, UK

## Abstract

The atomic geometries and transition levels of point defects and substitutional dopants in few-layer and bulk black phosphorus are calculated. The vacancy is found to reconstruct in monolayer P to leave a single dangling bond, giving a negative U defect with a +/− transition level at 0.24 eV above the valence band edge. The V^−^ state forms an unusual 4-fold coordinated site. In few-layer and bulk black P, the defect becomes a positive U site. The divacancy is much more stable than the monovacancy, and it reconstructs to give no deep gap states. Substitutional dopants such as C, Si, O or S do not give rise to shallow donor or acceptor states but instead reconstruct to form non-doping sites analogous to DX or AX centers in GaAs. Impurities on black P adopt the 8-N rule of bonding, as in amorphous semiconductors, rather than simple substitutional geometries seen in tetrahedral semiconductors.

There has been great interest in graphene as a 2-dimensional material with a high mobility^1^. Unfortunately, for field effect transistor (FET) applications, it has no band gap. Another family of 2-D materials, the transition metal dichalcogenides (TMDs) such as MoS_2_ and WSe_2_, do have a band gap and are therefore useful for FETs^2^,^3^,^4^. However, both their valence and conduction bands are formed from transition metal d orbitals, so that the carrier mobility is not so high, ~200 cm^2^V^−1^s^−1^, much less than that of graphene^4^,^5^. In addition, their device mobilities may be limited by contact resistance problems^6^,^7^,^8^,^9^. Therefore, there is a desire for a stable 2D semiconductor with a band gap, large carrier mobility and ability to form good contacts with standard electrode materials.

Black phosphorous (b-P) has recently been proposed as such a 2D system^10^,^11^,^12^,^13^,^14^,^15^,^16^,^17^,^18^,^19^,^20^,^21^,^22^,^23^,^24^,^25^,^26^,^27^. It has a buckled layer structure with 3-fold covalent bonding within each layer and van der Waals bonding between layers. In contrast to graphene, the lack of centre of symmetry means that b-P has a band gap. The monolayer has a direct band gap of 1.5eV, while the bulk band gap is about 0.3eV^10^,^11^. Its band edges are p states, so its carrier mobilities should be higher than those of MoS_2_ because p states are less sensitive to localisation than d states^12^,^13^. Field effect mobilities as high as 1000 cm^2^V^−1^s^−1^ have been reported^10^,^11^,^12^,^13^,^14^ while predictions of hole mobilities up to 10,000 cm^2^ V^−1^ s^−1^ have been given^15^,^16^,^17^,^18^. Being an elemental semiconductor, its defects might be simpler than those of binary compounds such as the TMDs. It is therefore important to determine electronic properties relevant to device performance, such as those of point defects that could trap carriers and limit field effect mobilities, or whether substitutional dopants can give shallow states to vary carrier concentrations and lower contact resistances.

Here, we calculate the band structures of few-layer black phosphorus. The energy gaps for monolayer and bulk are well reproduced. The electronic structure of the monovacancy is found to give rise to defects near the valence band whose configuration varies with charge state. The divacancy is found to have much lower formation energy than the monovacancy, and it reconstructs to give no deep gap states. Substitutional dopants are generally found to introduce deep defects or compensated bonding configurations, so that active shallow substitutional doping is difficult. In many ways the defect behaviour resembles that in amorphous semiconductors.

## Defect-free system

**Figure 1:**
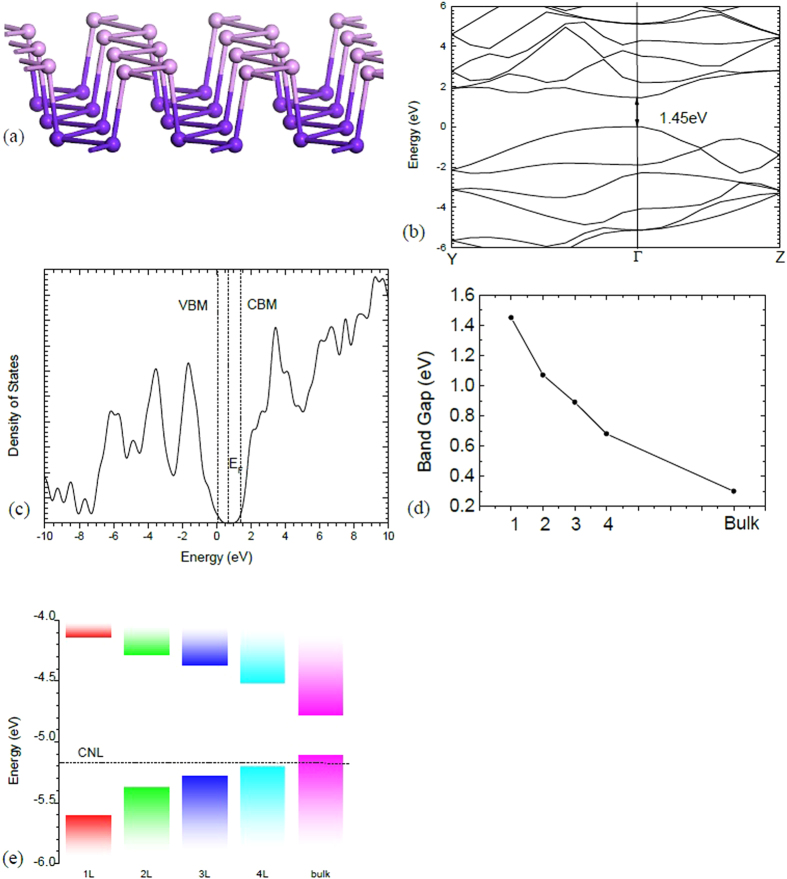
(**a**) Puckered layer structure of black phosphorus, showing our color scheme to differentiate atoms in the upper and lower parts of a layer. (**b**) Band structure of monolayer black P, (**c**) Total density of states. (**d**) Band gap versus number of layers in few-layer black P. CNL is the charge neutrality level. (**e**) band alignment vs number of layers.

The black P lattice consists of puckered layers, as shown in Fig. 1(a) with puckering along the Ox or ‘armchair’ direction, and zigzag bonding along the Oy direction^28^. This structure allows considerable mechanical flexibility and very large yield strains. Along the zigzag and particularly the armchair direction, strain is taken up in bond angle changes rather than bond-stretching. This leads to low elastic moduli, as bond-bending forces are less than bond-stretching forces. The ‘hinged’ structure also leads to a negative Poisson’s ratio^28^,^29^,^30^,^31^. These interesting properties also affect the defect properties. There is also a great contrast to the defect properties of graphene, where the bond-bending force constants of carbon are proportionately the highest of all group IV elements.

For the defect-free properties, the lattice constants are relaxed in both the generalised gradient approximation (GGA) and in the hybrid functional, and the band structure is calculated for the relaxed structure. The results are compared to experiments in Table 1. Figure 1(b–c) shows the band structure of the single layer and its density of states in screened exchange (sX). The valence band maximum (VBM) is set to be 0 eV in this figure. The calculations find that monolayer b-P has a direct gap of 1.45eV, while bulk b-P has a gap of 0.30eV. Both are in good agreement with previous experiments and calculations^16^,^17^,^18^,^19^,^20^,^21^,^22^. The PBE-style generalized gradient approximation gives a band gap of 1.0 eV for monolayer and a negative band gap for bulk b-P. Thus, the hybrid functional is necessary for electronic structure calculations of b-P.

Exciton energies are generally small in bulk 2D materials, but can become large for monolayer 2D materials, because of the lower screening^32^,^33^. A recent GW calculation of b-P suggests a large exciton energy of 0.5eV^18^. Interestingly, the hybrid functional is expected to give the optical gap rather than the quasi-particle gap in 2D materials^34^. Compared to the GW results, the flat dispersion curve at the conduction band maximum (CBM) near Γ along Y-Γ is fully reproduced. Moreover, the second lowest point at CBM is in between Y and Γ, which could be further lowered by applying strain.

Figure 1(d) shows the variation of the minimum gap at Γ with the number of layers, in good agreement with recent experiments^35^,^36^. The band gap E decreases as the number of layers N increases according to a power law^11^,^22^, E(N) = E_B_ + A/N^n^, where n is an index close to 1, E_B_ is the bulk band gap and A = E(1) − E_B_. The band gap decreases more slowly with N than it does in MoS_2_. The 4-layer b-P still has a band gap two times larger than the bulk value.

The charge neutrality level (CNL) is a useful reference energy that can be used to align band edges of different semiconductors and calculate Schottky barrier heights^37^,^38^. It can be calculated from the density of states (DOS). It is found to lie at 0.36 eV above the valence band edge in the monolayer case, and within the valence band for bulk b-P. We used the CNL to align the band edges of b-P for different layer numbers, as shown in Fig. 1(e). We see that the valence band maximum (VBM) changes slightly less than the conduction band minimum (CBM), whereas Cai *et al.*^22^ found the reverse.

## Monovacancies

**Figure 2:**
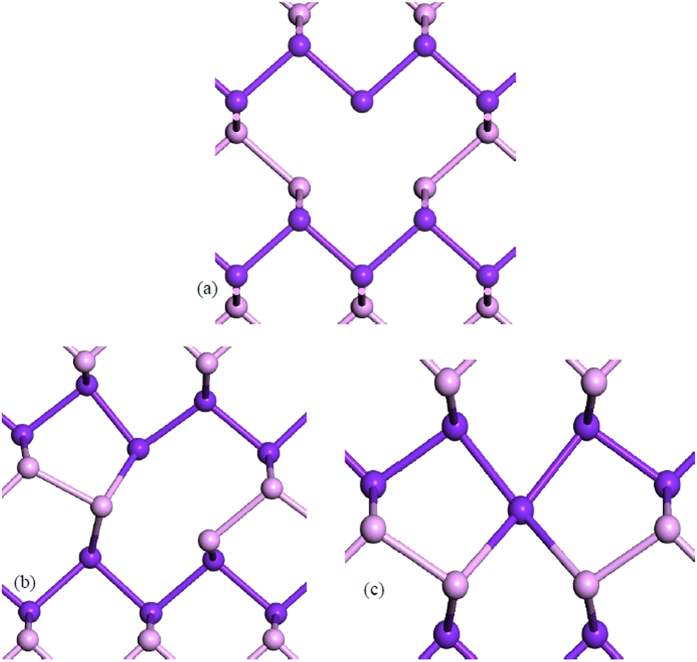
The single vacancy structure in monolayer black phosphorus. (**a**) ideal, unreconstructed, (**b**) reconstructed V^+^ vacancy and (**c**) reconstructed V^−^ vacancy.

Now consider the defect properties. Figure 2(a) shows the ideal (unrelaxed) monovacancy in monolayer b-P. Removing one P atom leaves a dangling bond (DB) on the three adjacent P sites. Because of the lattice flexibility, the vacancy can reconstruct so that two of the DBs rebond together, as in Fig. 2(b), leaving one DB. There are two choices for rebonding, as there are two DBs on ‘up’ sites and one on ‘down’ sublayer sites, or vice-versa. The rebonding occurs between ‘up’ and ‘down’ sites, so that one defect atom now bonds to two atoms on the opposite sublayer. The reconstruction leaves adjacent 9-fold ring and 5-fold ring of bonds, Fig. 2(b). This leaves one DB which can be charged +1, 0, or –1 according to its occupancy of 0, 1 or 2 electrons.

**Figure 3:**
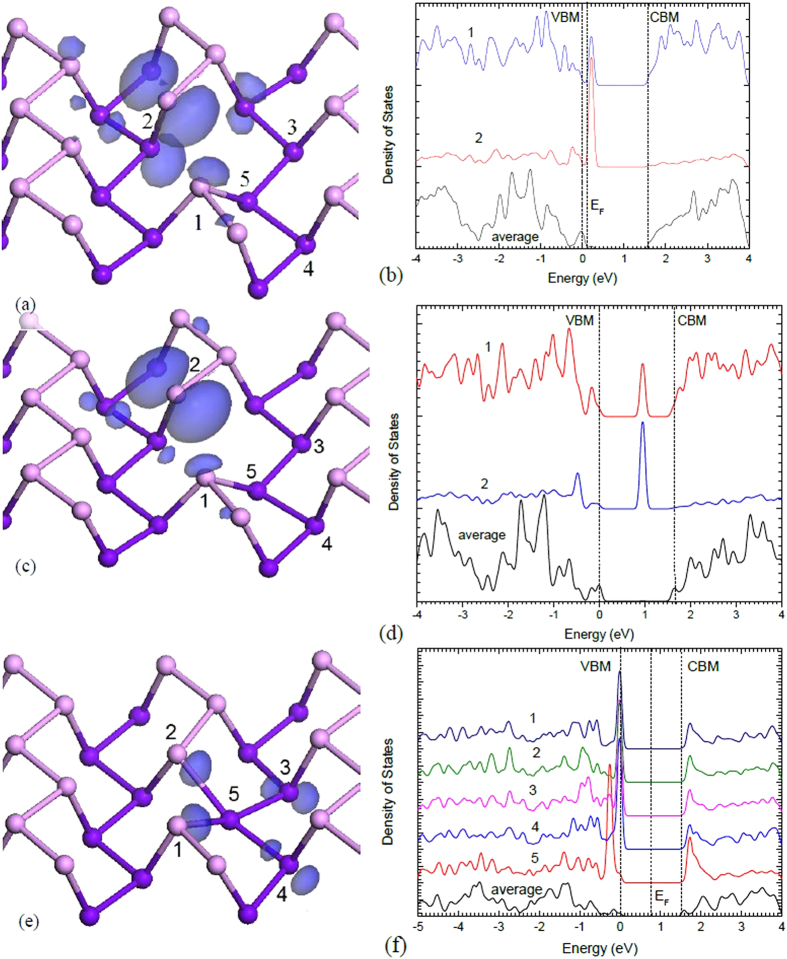
(**a,c,e**) Atomic geometries and (**b,d,f**) partial density of states (PDOS) of the vacancy in its +1, 0 and –1 charge states. Site numbering for the PDOS plots and the description of the 3-center bonding are given in **(a,c,e**).

Figure 3(a) shows the relaxed geometry and the DB defect orbital in the V^+1^ state, and Fig. 3(b) shows the partial density of states on the defect and neighbouring atoms. The defect orbital is largely localised at the DB and it has p-like symmetry. There is significant amount of the wavefunction localised on the 3-fold distorted P site formed by rebonding. The defect state creates an empty gap state at 0.2 eV above the VBM on monolayer b-P. The bond length of the newly formed bond is 2.352 Å, compared to a bulk bond length of 2.193 Å. From the atomic structure, we see that the defect induces a large local strain field around the vacancy, due to the small bulk modulus of black P. Our supercell convergence test shows that a large, 140-atom supercell is necessary for relaxation of this strain field

The neutral vacancy V^0^ adopts a similar geometry to V^+^, Fig. 3(c). The defect orbital is now half-filled; it gives rise to a filled spin-up state at -0.1 eV and an empty spin-down state near midgap, as seen in the PDOS in Fig. 3(d). The wavefunctions of the gap states for V^+^ and V^0^ are quite similar.

**Figure 4:**
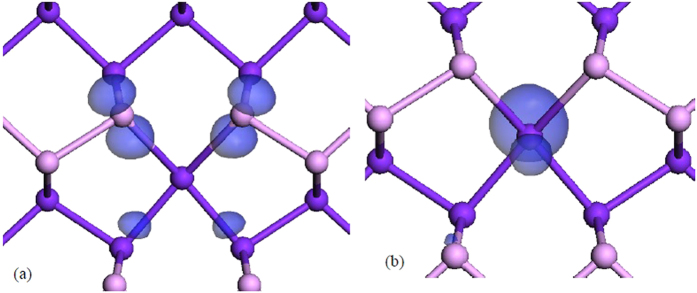
The two localised states associated with V^−^ that lie near the valence band edge.

The negative or V^−1^ state gives rise to a very different geometry. Here, the 2-fold ‘up’ atom rebonds to both 2-fold ‘down’ atoms, converting the ‘up’ atom into a 4-fold coordinated nearly-planar site, as shown from different viewing angles in Figs 2(c) and 3(e). The bonding of this site is now hypervalent. It gives rise to two peaks in the top of the valence band. There are four p orbitals on sites 1-4 of Fig. 3(e) directed towards the defect, each contributing 1 electron. On site 5, there are three p orbitals, also with 1 electron each. There is also one extra electron from the −1 charge, making a total of 8 p electrons. Two of these electrons enter a pπ state lying normal to the defect bonding plane. The other six enter three valence states. Two of these states consist of a p_x_ orbital on site 5 interacting with the p orbital combination ϕ = p_1_ + p_2_ − p_3_ − p_4_ on sites 1–4, and p_y_ on site 5 interacting with the combination ϕ = p_1_ − p_2_ + p_3_ − p_4_, both states lying deep in the valence band. The third state consists of the semi-bonding combination ϕ_s_ = p_1_ + p_2_ + p_3_ + p_4_, with no contribution from site 5. The state combination p_1_ − p_2_ − p_3_ + p_4_ is empty and lies in the conduction band. These defect wavefunctions associated with V^−^ are shown in Fig. 4, for comparison.

The side view of V^−^ shows that the central P atom is closer to the bottom layer, resulting in longer bonds to sites 1 and 2 (2.44 Å) and shorter bonds to sites 3 and 4 (2.31 Å). The four bonds are all longer than the average bond length in b-P of 2.19 Å.

The PDOS in Fig. 3(f) shows that the three-center bonds form a localised state at 0 to –0.01 eV, just below the valence band maximum (VBM). The pπ state lies slightly deeper at –0.25 eV below the VBM. The four neighbours of the central site, labelled as 1-4, are not equivalent. Atoms 1 and 2, 3 and 4 are equivalent respectively.

**Figure 5:**
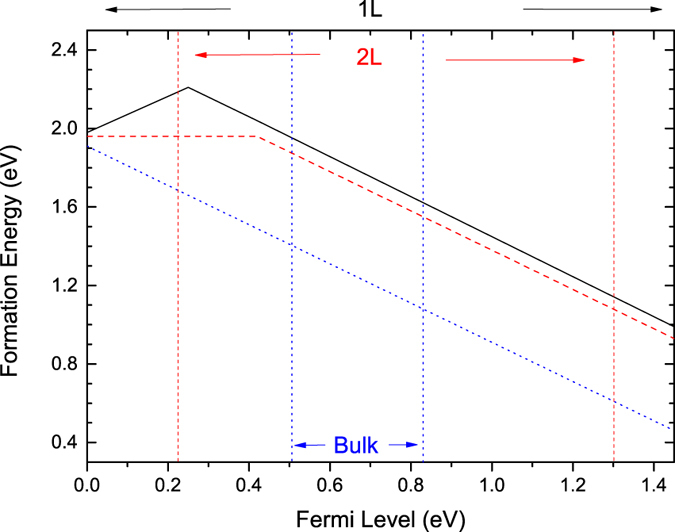
Formation energy of monovacancy vs. Fermi energy for monolayer, bilayer and bulk b-P. The +/− charge transition level lies at 0.24eV above VBM in monolayer b-P. In 2-layer b-P the 0/− level lies at 0.2 eV above its VBM. In bulk b-P, only the V^−^ state is stable.

The defect formation energy can be calculated for each charge state as a function of the Fermi energy as in Fig. 5. For monolayer b-P, we see that the V^−^ and V^+^ states are the stable ones, giving a +/− transition at 0.24 eV above the VBM. This makes the vacancy a negative U system with a small U of –0.18 eV. The paramagnetic neutral species is only metastable, and not so relevant for monolayer b-P. Note the dominance of the V^−^ charge state across most of the Fermi energy range. This shows the importance of considering all charge states, unlike in Liu *et al.*^23^.

Figure 5 also shows the formation energies for vacancies in multi-layer b-P. The formation energies are shown for two-layer b-P by red lines, and the band edges are also shown. The vacancy on the surface has an energy 0.24eV lower than the sub-layer site. The energy barrier between these two sites is less than 0.1eV according to transition state search so only the surface result is shown here. The V^−^ state has similar formation energy, but now the V^0^ state becomes a stable state. The V^0^ has basically the same geometry as the V^0^ or V^+^ in the monolayer case. Now the 0/−1 transition occurs at about 0.25 eV above the VBM. This is about 0.4 eV above the VBM reference of monolayer b-P. V is now a positive U defect in bilayer b-P.

Figure 5 also shows the data for bulk b-P, as blue lines. Here, the vacancy shows only the V^−^ charge state. This still has the 4-fold geometry as shown in Fig. 4. This means that the simple DB state lies below the VBM, which can cause unusual behavior. This would not happen for few layer b-P so that it is important to control the number of layers to be few. For a theory point of view, it illustrates the importance of relaxing the defect structure by a hybrid functional, not GGA, where the band gap is correctly given.

In graphene, for comparison, the vacancy undergoes a Jahn-Teller distortion which partially rebonds two of the DBs, leaving one main defect state near the Dirac energy^39^.

## Divacancy

**Figure 6:**
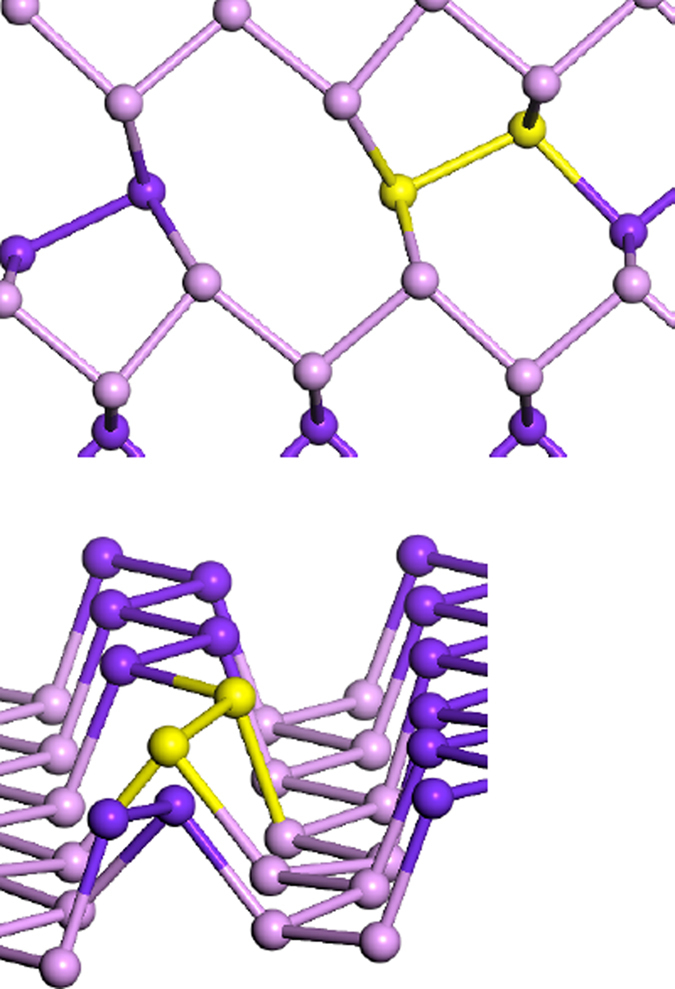
Two views of the reconstructed divacancy in monolayer b-P.

The divacancy in b-P is significant because it has a much lower formation energy than the monovacancy. We have studied different combinations of possible divacancy sites and Figs 6(a,b) shows the structure with the lowest formation energy after reconstruction, viewed from two different angles. The yellow bond shows one relevant bond in the different views. The lowest cost variant involves removing two atoms from the same sublayer, ‘up’ or ‘down’. The lattice then reconstructs bonding between ‘up’ and ‘down’ so that there are no DBs remaining. The divacancy is self-passivating. This divacancy creates one 8–fold ring and two adjacent 5-fold rings.

This strong rebonding leads to the low formation energy of 1.35 eV, which compares to 1.96 eV for *one* neutral monovacancy. Thus, there is a great energy gain if two monovacancies recombine into a divacancy.

We find that the divacancy gives no deep states in the gap. The defect acts like a tail-state in amorphous Si; the bond angle distortions create shallow defects close to the band edges and this will lead to a reduction in carrier mobility. Thus, vacancy defects are relatively passive in b-P if they can combine into divacancies, as noted by Liu^23^.

We can compare this defect to the divacancy in Si and graphene. In Si, the monovacancy has four DBs, these undergo Jahn-Teller reconstructions according to the occupation, but there are always four states in the gap. The divacancy in Si has slightly lower cost than two monovacancies. The divacancy formally has six DBs. There is a distortion leading to weak rebonding across the divacancy in Si but this is weak, because of the large separation. Thus, there remain still six defect related levels in the gap.

In graphene, there is more attempt at rebonding. The basic divacancy creates a 5:8:5-fold ring network^40^. However, this leads to bond angle strain, because of the strong bond-bending force constants. Thus the lattice creates Stone-Wales rearrangements, which lower the strain but create more odd membered rings around the divacancy. The 5:8:5 defect thereby becomes a 5:5:5 – 7:7:7 ring defect or further restructuring^40^.

## Dopants

**Figure 7:**
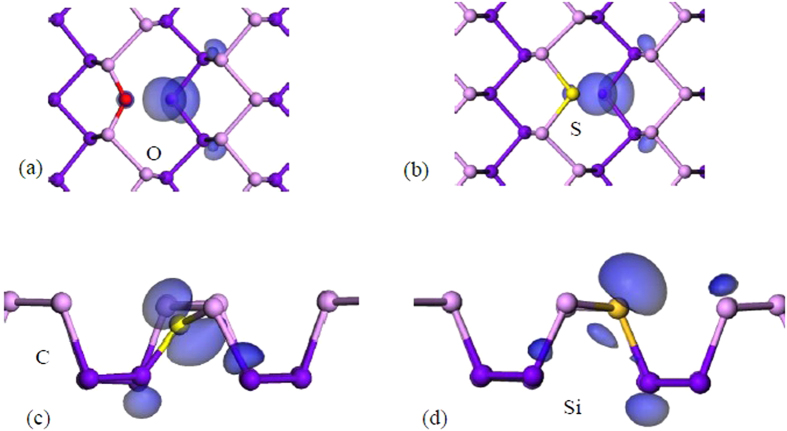
(**a**–**d**) Defect orbitals due to substitutional O, S, C, and Si. The dopant atom is labelled with different colors.

**Figure 8:**
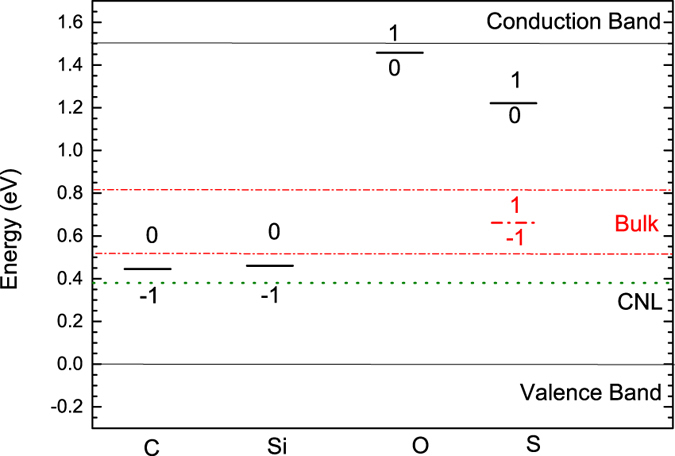
Summary of transition states of dopants in b-P. C and Si form deep acceptors. O forms a moderately shallow donor, while C forms a deep donor.

Substitutional doping is a very important factor to find a way to stably shift the Fermi energy, such as to lower contact resistances in devices. Interstitial doping is a less useful method, as in this case the ions can easily diffuse away. As P is from column V, we consider the behavior of O and S as substitutional donors from column VI, and C and Si as acceptors from column IV, Fig. 7. Our calculated defect levels are summarized in Fig. 8. For monolayer b-P, Fig. 7(a–d) show that the substitutional O and S sites both reconstruct in monolayer P, so that one O-P or S-P bond breaks, making divalent O or S sites and a P dangling bond. This means that there is no donor state. For multi-layer b-P, the substitutional S always has the broken S-P bond, with the S site being 2-fold bonded. In contrast the O site becomes 3-fold coordinated.

For substitutional C or Si, the sites relax towards a planar geometry. For the case of C, the site becomes planar. This leaves an empty π state in the upper band gap. As this acceptor state is not near the valence band, there is no doping effect and E_f_ lies near mid gap below this empty state. In the case of Si, the relaxation is less, and the site retains some sp^3^ character. It leaves an empty Si dangling bond, which lies in the lower part of the gap, but it is not a shallow level. It is reminiscent of the 3-fold Si at a Si(110) surface. There is some doping character, with E_f_ lying near the valence band edge.

Overall, the low dimensionality of black P has allowed the standard substitutional dopants to relax, and exert their preferred 8-N coordination (O, S) as occurs in amorphous semiconductors^41^,^42^, or form defect states, so there is no doping effect. This is also similar to the response of dopants in wider gap semiconductors like GaAs where donors can form non-doping DX centers and acceptors can form AX centers^43^.

These results show a contrast to conventional tetrahedrally-bonded semiconductors. There, the cohesive energy peaks in the middle of the Periodic Table at column IV because of the stability of sp^3^ bonding. This and the three dimensional bonding inhibit symmetry lowering to make the non-doping geometries. On the other hand, 2D semiconductors gain mobility due to restrictions on carrier scattering, but they loose out on the ability to control the Fermi energy by doping. In some ways Mo and W dichalcogenides do retain an ability to form stable substitutional sites despite lower dimensionality because Mo and W sites have high cohesive energy. In contrast, b-P has lower cohesive energy than neighboring Si, and there is nothing to enforce true substitutional geometries and a doping response.

It is interesting to see how the reconstructions can vary with the number of layers. The driving force for reconstruction and passivation for dopants increases with the band gap, as in DX centers^44^. Thus, a simple doping response is more likely for few layer b-P than in the monolayer case.

## Summary

In conclusion, the electronic structure of monolayer, few-layer, and bulk black phosphorus has been calculated by the sX hybrid functional. The band structure agrees well with experiments. The P vacancy is found to be a p-type defect lying 0.24eV above the VBM. The P vacancy is a negative U defect with +/− transition state for the monolayer P. For few layer and bulk P, the neutral state becomes more stable, and the vacancy becomes a positive U defect. The change of charge state will introduce significantly distortion to the lattice. The possible doping of O, S, C, and Si is considered. None of them could give p-type doping. Overall, the lack of doping effect means that black P might need to use work function control for best contacts, and not rely on doping to optimize contact resistances.

## Methods

The calculations are carried out using the plane-wave, density functional code CASTEP^45^. Norm-conserving pseudopotentials are used with a cut-off energy of 500 eV. Corrections to the DFT treatment of van der Waals interactions are taken into account empirically by using Grimme’s method^46^,^47^.

Standard density functional theory (DFT) has previously been applied to b-P, but it gives either a small or negative band gap for bulk b-P. Thus, we use the screened exchange (sX) hybrid density functional^48^ to correct the band gap errors of standard DFT. Hybrid functionals have been successfully applied to the electronic and defect calculations in other 2D systems such as graphene and MoS_2_^32^.

We use a supercell model for the mono- and few-layer systems, with a vacuum layer of 25 Å. This is checked to be a good approximation for a 2D slab system. The defect calculations used 140-atom supercells for the few-layer and bulk cases. The minimum distance between mirror image defects is 22 Å. Large supercells are needed for the defect calculations in b-P because of the softness of its lattice, which leads to relatively long-range structural relaxations around defects. A 2 × 2 × 2 Monkhorst-Pack k-point mesh is used for reciprocal space integration in all supercells. The above parameters lead to an energy convergence to less than 0.02eV.

For defects, the charge transition states are calculated using the supercell method. Corrections for defect charges and band occupations are applied as in ref. [49]. The total energy of the perfect supercell (*E*_*H*_) and the supercell with defect (*E*_*q*_) are calculated for different charge states. The defect formation energy *H*_*q*_ is then found from


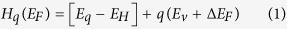


where *q*E_v_ is the change in Fermi energy when charge *q* is added. No extra correction is needed for a two-dimensional calculation.

## Additional Information

**How to cite this article**: Guo, Y. and Robertson, J. Vacancy and Doping States in Monolayer and bulk Black Phosphorus. *Sci. Rep.* **5**, 14165; doi: 10.1038/srep14165 (2015).

## Figures and Tables

**Table 1:** Lattice constants compared with previous results.

Å		a	b	c
Bulk	Experiment [30]	10.47	3.31	4.37
	This work	10.794	3.291	4.379
Monolayer	Other [10]		3.32	4.58
	This work		3.279	4.521
